# A comprehensive survey of copy number variation in 18 diverse pig populations and identification of candidate copy number variable genes associated with complex traits

**DOI:** 10.1186/1471-2164-13-733

**Published:** 2012-12-27

**Authors:** Congying Chen, Ruimin Qiao, Rongxing Wei, Yuanmei Guo, Huashui Ai, Junwu Ma, Jun Ren, Lusheng Huang

**Affiliations:** 1 Key Laboratory for Animal Biotechnology of Jiangxi Province and the Ministry of Agriculture of China, Jiangxi Agricultural University, Nanchang, 330045, China; 2 Nanchang Working Canine Base, Nanchang, 330045, China

**Keywords:** Copy number variation, Copy number variable gene, Complex trait, QTL, Pig

## Abstract

**Background:**

Copy number variation (CNV) is a major source of structural variants and has been commonly identified in mammalian genome. It is associated with gene expression and may present a major genetic component of phenotypic diversity. Unlike many other mammalian genomes where CNVs have been well annotated, studies of porcine CNV in diverse breeds are still limited.

**Result:**

Here we used Porcine SNP60 BeadChip and PennCNV algorithm to identify 1,315 putative CNVs belonging to 565 CNV regions (CNVRs) in 1,693 pigs from 18 diverse populations. Total 538 out of 683 CNVs identified in a White Duroc × Erhualian F_2_ population fit Mendelian transmission and 6 out of 7 randomly selected CNVRs were confirmed by quantitative real time PCR. CNVRs were non-randomly distributed in the pig genome. Several CNV hotspots were found on pig chromosomes 6, 11, 13, 14 and 17. CNV numbers differ greatly among different pig populations. The Duroc pigs were identified to have the most number of CNVs per individual. Among 1,765 transcripts located within the CNVRs, 634 genes have been reported to be copy number variable genes in the human genome. By integrating analysis of QTL mapping, CNVRs and the description of phenotypes in knockout mice, we identified 7 copy number variable genes as candidate genes for phenotypes related to carcass length, backfat thickness, abdominal fat weight, length of scapular, intermuscle fat content of logissimus muscle, body weight at 240 day, glycolytic potential of logissimus muscle, mean corpuscular hemoglobin, mean corpuscular volume and humerus diameter.

**Conclusion:**

We revealed the distribution of the unprecedented number of 565 CNVRs in pig genome and investigated copy number variable genes as the possible candidate genes for phenotypic traits. These findings give novel insights into porcine CNVs and provide resources to facilitate the identification of trait-related CNVs.

## Background

Copy number variation (CNV), a major type of structural variants, is defined as DNA segments that vary from one kilobase to several megabases in length and present at variable copy numbers in comparison with a reference genome [1,2]. Following the completion of pig whole-genome sequencing, genome-wide polymorphisms including CNV, SNP and deletion/insertion will be well annotated in the near future. Currently, Porcine 60K SNP BeadChips are commercially available for genome-wide analyses of 62,163 SNPs. However, a comprehensive study of genome-scale CNVs in pigs remains unexplored.

Tilling oligonucleotide array and comparative genomic hybridization (CGH) array have been commonly used to detect whole-genome CNVs. In recent years, whole-genome SNP genotyping arrays offer alternative methods for CNV detection [3]. Computer programs like PennCNV [4], QuantiSNP [5], cnvPartition (Illumina) and CNV Workshop [6] have been developed to identify CNVs from SNP array data. The unique ability to integrate family relationships from parent-offspring trios, total signal intensity and allelic intensity ratio at each SNP marker, and the allele frequency of SNP makes PennCNV algorithm have a moderate power and the lowest false positive rate [4]. Winchester *et al.* (2009) reported that PennCNV is the most accurate program in the prediction of CNVs for the Illumina’s platform by comparing different algorithms for CNV detection [7].

CNVs have been commonly identified in humans, rats, dogs, cattle and horse, and occupy about 3.7%, 1.4%, 4.2%, 4.6% and 3.6% of their assembled genome, respectively [8-12]. It has been estimated that CNVs account for at least 17.7% of heritable variation of gene expression in a variety of ways including gene dosage effects, disruption of gene coding region and deletion or duplication of regulatory elements [13]. In humans, SNP-tagged CNVs are enriched for expression quantitative trait loci (eQTL) [14]. CNVs have been confirmed to associate with Mendelian diseases and complex genetic disorders in humans, such as schizophrenia [15], body mass index [16], Crohn’s disease [17] and intellectual disability and various congenital defects [18]. Similarly, in livestock, more and more studies evidenced that CNVs play causative effects on phenotypic variations, such as CNV in intron 1 of *SOX5* causing the pea-comb phenotype in chickens [19], a 4.6-kb intronic duplication in *STX17* for hair greying and melanoma in horses [20], duplication of *FGF3*, *FGF4*, *FGF19* and *ORAOV1* resulting in hair ridge and predisposition to dermoid sinus in Ridgeback dogs [21], and CNV and missense mutations of the agouti signaling protein (*ASIP*) gene leading to different coat colors in goats [22]. But in pigs, few such examples have been reported at present. *KIT* is the first pig gene that has been confirmed that gene duplication and a splice mutation leading the skipping of exon 17 are responsible for the dominant white phenotype and peripheral blood cell [23,24].

Until now, to our knowledge, there are only three studies on pig CNV discovery. Fadista *et al.* (2008) found 37 CNVRs across chromosome (SSC) 4, 7, 14 and 17 using a custom tilling oligonucleotide array [25]. Tang *et al.* (2010) investigated the CNV distribution on SSC7 and SSC8 by CGH array [26]. More recently, Ramayo-Caldas *et al.* (2010) detected 49 CNVRs in porcine autosomal chromosomes in 55 animals from an Iberian × Landrace cross with Porcine SNP60 BeadChip [27]. However, the distribution of CNVs in large scale and diverse pig populations remains largely unknown.

We herein used Porcine SNP60 BeadChip and PennCNV algorithm to identify porcine autosomal CNVs in 1,693 animals from 18 populations, and analyzed the CNV distribution in pig genome and different populations. We compared the identified CNVs with the reported porcine CNV call sets and investigated the copy number variable genes. Especially, we used a large scale White Duroc × Erhualian F_2_ intercross, in which the QTL were mapped for 422 traits [28]. The intercross allowed us to systematically investigate the effects of pig CNVs on phenotypic variations.

## Results and discussion

### CNV discovery, distribution and validation

Experimental samples were recruited from 18 pig populations including 10 Chinese indigenous breeds with different geographical origins, 2 Western commercial breeds, 1 wild boar and 5 F_2_ resource populations (Table 1). The Porcine SNP60 BeadChip data that passed quality control in a panel of 1,693 animals were included in the analysis of CNVs by PennCNV. We used a calling criterion of spanning three or more consecutive SNPs and standard deviation of log R Ratio ≤ 0.35. The chromosomes X and Y were excluded from our analysis. As a result, we totally identified 2,122 putative CNVs in 1,327 individuals including 971 population-specific CNVs. These CNVs are located in all 18 autosomes with a mean size of 223.51 kb ranging from 50.02 kb to 5.64 Mb. The predicted status for the CNVs was 1,149 (54.15%) for deletion, 964 (45.43%) for duplication and 9 (0.42%) for regions with either deletion or duplication status according to different animals (Table 1).

**Table 1:** Identification of CNVs in 18 diverse pig populations

**Breed**	**Number of animals identified CNV**	**Number of CNVs**		**Status of CNVs**			**Average Size(kb)**
		**Total**	**Unique**	**Gain**	**Loss**	**Gain/Loss**	
White Duroc × Erhualian F_2_ intercross	752	683	456	370	305	8	227.21
Bamaxiang × Erhualian F_2_ intercross	77	92	30	41	51	-	184.42
Rongchang × Erhualian F_2_ intercross	87	159	48	65	94	-	218.72
Shazilin × Erhualian F_2_ intercross	156	254	149	153	101	-	240.92
Tongcheng × Erhualian F_2_ intercross	51	104	22	53	51	-	218.52
Bamaxiang	15	66	35	7	59	-	206.91
Dongshan	9	35	12	12	23	-	215.13
Duroc	10	124	52	13	111	-	220.26
Erhualian	16	48	6	16	32	-	232.96
Jinghua	14	75	40	19	56	-	191.21
Minzhu	15	46	6	22	23	1	230.86
Rongchang	63	180	64	90	90	-	194.64
Shanggao Two-End-Black	10	31	6	7	24	-	170.79
Shazilin	8	34	5	12	22	-	144.51
Sutai	10	57	13	22	35	-	206.23
Tongcheng	15	50	12	23	27	-	153.23
White Duroc	3	23	0	6	17	-	183.52
wild boar	16	61	15	33	28	-	141.83
Total	1,327	2,122	971	964	1,149	9	-
Average	-	117.89	53.94	53.56	63.83	0.50	223.51

**Figure 1:**
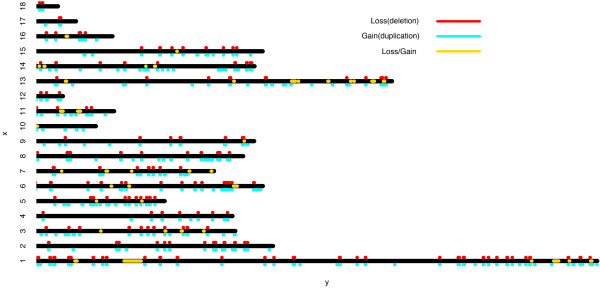
**Distribution of CNVRs detected in this study in sus scrofa reference genome assembly 10.2.** Black lines represent all 18 porcine autosomes. Red dots indicate duplicated CNVRs, while deleted CNVRs are highlighted in cyan and regions with both loss and gain are indicated in orange

Merging identical CNVs from all animals across breeds yielded 1,315 unique CNVs out of the 2,122 putative CNVs. CNVRs were determined by aggregating overlapping unique CNVs. The 1,315 unique CNVs were clustered into a set of 565 non-redundant CNVRs which encompassed about 143.03-Mb region equaling approximately 5.84% of the pig genome (Figure 1). These 565 CNVRs were all called in ≥ 2 individuals and included 261 loss, 225 gain and 79 both (Additional file 1: Table S1). The sizes of these CNVRs ranged from 50.39 kb to 8.10 Mb, with a median size of 252.71 kb (Additional file 1: Table S1). This size range was significantly different from that of detected by the CGH array (ranged from 1.74 to 61.92 kb) [25]. This difference may be explained by the relatively low coverage and the non-uniform distribution of SNPs in Porcine SNP60 BeadChip in the pig genome [29]. But we noted that the size range in this study was similar to that in Ramayo-Caldas *et al.* (2010) where CNVRs were also called with genotyping data from Porcine SNP60 BeadChip [27].

Just as the cases in human [11] and cattle [30], we found that the CNVRs were non-randomly distributed across the pig genome. Chromosome 4, for instance, has only 2.02% of sequences showing copy number variable, while chromosome 18 has > 18.01% of sequences with copy number variation. Several “hotspots” of copy number variation were obsevered in this study, such as SSC6: 138.40-145.74 Mb, SSC11: 43.58-46.62 Mb, SSC13:213.45-215.94 Mb, SSC14: 2.74-7.42 Mb and SSC17: 8.25-10.28 Mb. These regions contain clusters of four to six CNVRs, indicating CNV hotspots (Additional file 1: Table S1; Figure 1).

**Figure 2:**
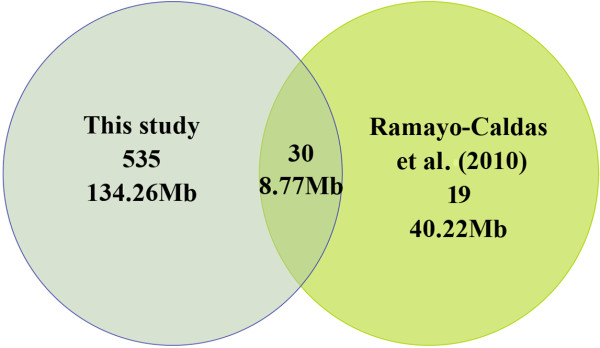
Comparison between 565 CNVRs identified in this study and a previously reported porcine CNV dataset in terms of count and length

The quality of our CNV calls was assessed in multiple ways. Our first assessment was a comparison against a previously reported porcine CNV dataset identified in 55 animals from an Iberian × Landrace cross with Porcine SNP60 BeadChip [27]. We found 30 CNVRs that overlapped with CNVRs in that dataset, accounting for 61.22% of their CNV calls (Figure 2). The 38.78% of unreplicated CNVRs is most likely due to different genetic background of pig populations in two studies, and certainly, false positive can not be excluded. For 683 CNVs detected in the White Duroc × Erhualian F_2_ resource population, we validated their Mendelian transmission using family information. Total 538 out of 683 CNVs fit Mendelian transmission (78.77%). Finally, a total of 7 CNVRs were randomly selected for validation by quantitative real time PCR assays (CNVR329, 361, 419, 482, 502, 509 and 531; Additional file 2: Table S3). Except CNVR509 for which none of 6 animals were confirmed for this CNVR, we validated the other 6 CNVRs (Additional file 3: Figure S1).

### Porcine CNV frequencies among breeds

Like CNV frequencies in humans [31], CNV numbers differ greatly among different pig populations. The average number of CNVs per population was 117.89, ranging from 23 (White Duroc) to 683 (White Duroc × Erhualian F_2_ intercross). The most number of CNVs per sample was detected in Duroc pigs (12.40 CNVs per animal on average), in comparison with the least number of 0.91 CNVs per animal in the White Duroc × Erhualian F_2_ intercross (Table 1).

Of the 565 CNVRs, only 20 CNVRs were detected in more than 50% of populations (9 populations). Similar to the finding in humans [31], most CNVRs (72.87%) were restricted to one population. This should be due to sampling variances or the fact that they were recent evolution events. Among 18 diverse populations, the largest number of 310 CNVRs was identified in the White Duroc × Erhualian F_2_ intercross (Additional file 4: Table S2). It is most likely due to the fact that this population was a cross between European and Asian divergent breeds. Of these 310 CNVRs, 215 were unique to this resource population. As two Western pig breeds (Duroc and White Duroc) used in this study, we found that all 5 CNVRs identified in White Duroc were included within 42 CNVRs detected in Duroc. It is consistent with the fact that White Duroc is one of specialized lines of Duroc. Total 26 CNVRs were unique to the two Western breeds. Among Chinese indigenous pig breeds, the most number of 30 CNVRs was identified in both Jinhua and Shazilin. Moreover, Jinhua had the highest average number of CNVs per individual (5.36 per individual; Additional file 4: Table S2).

### Gene content of CNV regions

The BioMart gene database based on the Sscrofa 10.2 reference genome assembly [32] was used to retrieve genes within the detected CNVRs. A total of 1,764 transcripts were annotated within 320 out of 565 putative CNVRs, including 1,546 transcripts completely located within the CNVRs and 218 transcripts overlapping with the CNVRs. These 1,764 transcripts were composed of 1,587 protein coding genes, 37 pseudogenes, 1 retrotransposed genes, 42 miRNAs, 7 rRNAs, 36 snoRNAs, 47 snRNAs and 7 miscRNAs. No annotated transcripts were identified within the other 235 CNVRs. Of the 1,587 protein coding genes, 1,055 genes were well annotated in pigs including 634 genes that have CNVs in the human genome (Additional file 5: Table S4) [33]. The average gene number per Mb in CNVRs was 13.26, 14.74 and 6.01, respectively, for gain, loss and gain/loss. However, compared to the average gene number per Mb in whole genome, the CNVRs have higher gene density (11.21 vs. 8.37). This result was consistent with the findings in other species in which a higher gene content was discovered in CNVRs [9,29,30].

**Figure 3:**
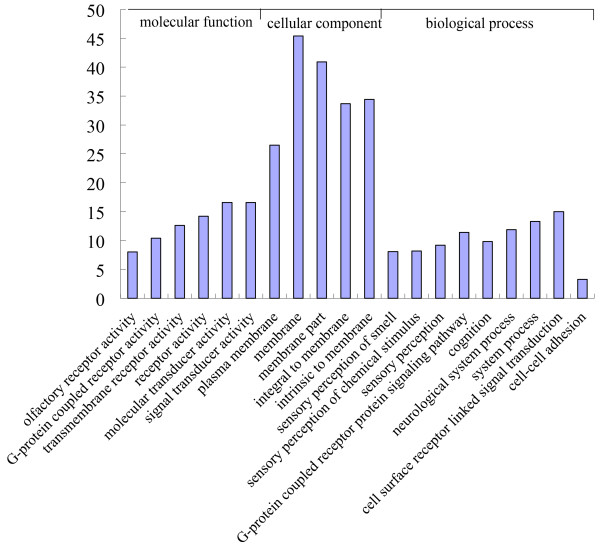
**Functional categories of CNV-related genes by gene ontology analysis.** The bar plot represents the percentage of gene counts within each GO category. All functions or processes listed have enrichment of corrected *P* values < 0.05

CNV-associated genes or copy number variable genes have a wide spectrum of molecular functions and provide a resource for investigating the biological relationship of CNVs with the genetic basis of phenotypic variations. We performed the gene ontology (GO) analysis by querying each copy number variable gene into the records of the GO database [34]. Similar to GO annotation of CNV-associated genes in humans and rats [9,30], the main terms of molecular function are related to olfactory receptor activity, G-protein coupled receptor activity, transmembrane receptor activity, receptor activity, molecular transducer activity and signal transducer activity. In the cellular component category, the most significant term was plasma membrane (corrected *P* = 2.70 × 10^-4^). Among GO biological processes, the most overrepresented one was sensory perception of smell (*P* = 5.50 × 10^-18^, Figure 3). Most these terms relate to the olfactory receptors. This may be due to the fact that total 84 *olfactory receptor* genes were included in the detected copy number variable genes.

We found that many CNV-associated genes appear to be certain gene clusters or families, such as *olfactory receptor family*, *solute carrier family*, *apolipoprotein gene family*, *myosin gene family*, *CD gene family*, *cytochrome C oxidase gene family*, *interleukin gene family*, *protocadherin gamma gene cluster*, *beta defensin protein family*, *neuroblastoma breakpoint family*, *zinc finger protein family* and *ring finger protein family* (Additional file 5: Table S4). Some of these gene families have been well characterized and play important roles in biological processes [27,35]. For example, *olfactory receptor family* is the most well characterized CNV-related genes in humans [35]. Over 400 human *olfactory receptor* genes are reported to be variable in copy number [36]. In this study, total 84 *olfactory receptor* genes were located within CNVRs. More than 15 members of *solute carrier family* were detected in CNVRs in this study. *Solute carrier family* encodes membrane transport proteins including over 300 members organized into 51 families. The family proteins play important roles in transportation and exchange of ion, amino acid and other substance which take part in important biological process [37].

### Identification of copy number variable genes as potential candidate genes for complex traits

The potentially disruptive effect of CNVs on gene expression, structure and function indicates that CNVs are likely to contribute to phenotypes. Before this study, a genome-wide QTL mapping for traits of blood parameter [38], meat quality [39], fatness [40], growth [40], reproduction [41,42], immune capacity [43] and body conformation [44] was carried out in the White Duroc × Erhualian F_2_ resource population and all QTL have been published and deposited in the pig QTL database [45]. In this study, all QTL above 5% genome-wide significance level were chose to match with CNVRs identified in the White Duroc × Erhualian F_2_ resource population. By integrating analysis of QTL mapping, CNVRs and the description of phenotypes in knockout mice, we identified 7 CNV genes including *ANP32B*, *BSCL2*, *LTBP3*, *GDF3*, *GYS1*, *KIT* and *CAV1* as potential candidate genes for phenotypes related to carcass length, backfat thickness, abdominal fat weight, length of scapular, intermuscle fat content of logissimus muscle (LD), body weight at 240 day, glycolytic potential of LD, mean corpuscular hemoglobin (MCH), mean corpuscular volume (MCV) and humerus diameter (Table 2).

**Table 2:** **Identification of potential candidate CNV genes for complex traits in the White Duroc × Erhualian F**_**2**_**population**

**CNVR ID**	**CNVR region**	Trait of overlapped QTL*	**CNV gene**	**Phenotype in knockout mice**	**Reference**
CNVR41	chr1: 267,977,629-268,124,492	carcass length	ANP32B	decreased body size	[39,46]
CNVR62	chr2: 8,244,738-8,884,302	Backfat thickness	BSCL2	decreased subcutaneous adipose tissue	[40,47]
		abdominal fat weight	BSCL2	decreased retroperitoneal fat pad weight	[40,47]
CNVR61	chr2: 5,766,853-6,201,646	length of scapular	LTBP3	decreased length of long bones	[44,48]
CNVR169	chr5: 65,533,763-65,810,346	intermuscle fat content of LD	GDF3	decreased white adipose tissue amount	[39,49]
		body weight at 240 days	GDF3	abnormal developmental patterning	[40,50]
CNVR199	chr6: 49,802,217-50,638,891	Glycolytic potential of LD	GYS1	decreased skeletal muscle glycogen level	[39,51]
CNVR268	chr8: 43,425,758-43,955,459	mean corpuscular volume	KIT	increased mean corpuscular volume	[43,52]
		mean corpuscular hemoglobin	KIT	decreased hemoglobin content	[43,53]
				diluted coat color	[54]
CNVR560	chr18: 27,001,689-32,149,496	Humerus diameter	CAV1	increased bone size and stiffnes	[44,55]

* QTL identified in the White Duroc × Erhualian resource population.

We further chose *KIT* as a proof-of-principle example as the confirmed association between *KIT* duplication and MCH and MCV. Previous studies have confirmed that *KIT* regulatory mutations including the gene duplication and splice mutation are responsible for the dominant white phenotype in pigs and have pleiotropic effects on peripheral blood cell measures in Western commercial pigs [23,24]. A significant QTL for MCH and MCV at day 240 was detected at SSC8: 43,550,231 in the White Duroc × Erhualian F_2_ resource population [43], which fell within the genomic region of CNVR268 (SSC8: 43,425,758-43,955,459; Table 2). More than 378 F_2_ animals from the intercross inherited this CNV variant. Two genes of *KIT* and *KDR* were located within this CNVR. The *KIT* knockout mice exhibited phenotypes of increased mean corpuscular volume, decreased hemoglobin content and diluted coat color [54]. Furthermore, association analysis showed that CNV268 was significantly associated with MCH and MCV in the White Duroc × Erhualian F_2_ resource population (*P* = 1.17 × 10^-5^). And if we included the CNV268 as fixed effect in QTL mapping, the QTL on SSC8 for MCH and MCV was never detected again.

Moreover, the result obtained in this study was also consistent with the causative relation between *KIT* duplication and dominant white coat color identified before [56]. The CNVR268 harboring *KIT* was detected only in the solid white breed White Duroc. It was absent in all other pigs from diverse populations having colored phenotypes. It is noteworthy that the CNVR was either not found in Chinese belt-like breeds including Bamaxiang, Dongshan, Shanggao, Jinhua, Shazilin and Tongcheng, or in Rongchang pigs with the white coat color. This was in agreement with our previous conclusions that the belt-like and white coat colors in Chinese pigs were not caused by the dominant white allele of *KIT*[57,58].

Although some identified copy number variable genes were not overlapped with our reported QTL, they have been reported to associate with complex traits in pigs, humans or mice. For instance, we detected an 836.67-kb CNV in SSC6: 49,802,217-50,638,891 in the White Duroc × Erhualian resource population. This region contains the alpha-1-fucosyltransferase (*FUT1*) gene. *FUT1* has been identified as a strong candidate gene encoding the intestinal *Escherichia coli* F18 receptor that determines susceptibility to oedema disease, post-weaning diarrhoea in Western piglets and total number of born piglets [59,60]. *APOE/C4/C2* gene cluster is located within a 427.46-kb CNV region on SSC6: 46,893,592-47,321,053. The *APOE/C1/C4/C2* gene cluster variation in humans is associated with plasma lipids, particularly low density lipoprotein (LDL) level and coronary heart disease [61]. CNVR128 (SSC3: 120,293,272-120,344,603) harbors *HADHA* and *HADHB*, encoding the mitochondria trifunctional protein (MTP) alpha- and beta-subunits, respectively. The *HADHAHADHB* deficient mice had a decreased weight gain and cardiac arrhythmias [62].

## Conclusions

In this study, we revealed the distribution of the unprecedented number of 565 CNVRs in 1,327 pigs from 18 diverse populations, and found that CNVRs were non-randomly distributed in the porcine genome. CNV numbers differ greatly among diverse populations. More than 72.87% of CNVRs were restricted to one population. The main functional categories of CNV-related genes were similar to those of in other mammals. With the QTL mapping data and the identified CNVRs in the White Duroc × Erhualian F_2_ intercross, and the description of phenotypes in knockout mice, we identified 7 copy number variable genes as potential candidate genes for porcine complex traits. These findings give novel insights into porcine CNVs and provide resources to facilitate the further identification of trait-related CNVs.

## Methods

### Animals

A total of 1,693 animals from 18 pig populations including 10 Chinese indigenous breeds, 2 Western commercial breeds, 1 wild boar and 5 F_2_ resource populations comprising White Duroc × Erhualian, Bamaxiang × Erhualian, Rongchang × Erhualian, Shazilin × Erhualian and Tongcheng × Erhualian were used in this study (Table 1). Thereinto, 1,021 animals were obtained from the large scale White Duroc × Erhualian F_2_ resource population in which two White Duroc boars and 17 Erhualian sows were crossed as founder animals to produce F_1_ animals, and 59 F_1_ sows were randomly mated with 9 F_1_ boars to generate 1,912 F_2_ individuals. Total 422 traits related to growth, meat quality, body composition, blood physiological and biochemical parameters, reproduction and immune capacity were well phenotyped in this intercross and genome-wide QTL mapping was carried out for these traits [38-44]. All animal procedures were conducted according to the guidelines for the care and use of experimental animals established by the Ministry of Agriculture of China.

### Genome-wide SNP genotyping

Genomic DNA was extracted from ear or spleen tissues with the routine phenol/chloroform extraction method. All 1,693 animals were genotyped with Porcine SNP60 BeadChip using the *Infinium HD Assay Ultra* protocol (Illumina Inc., San Diego, USA). The position of each SNP in the pig genome assembly (Sscrofa10.2) was determined by SOAP2 software [63]. The quality control of genotypes was performed with GenABEL procedure in R. The SNPs in sex chromosomes and those not mapped or mapped to multi-positions in the Sscrofa10.2 assembly were discarded. A final set of 5,2596 SNPs on 18 autosomes with a unique position in Sscrofa10.2 was used for further analysis.

### CNV calling

PennCNV was used to CNV calling. The software integrates a Hidden Markov Model (HMM) for high resolution copy number variation detection with whole-genome SNP genotyping data [4]. The signal intensity data of log R Ratio (LRR) and B allele frequency (BAF) were exported from Illumina BeadStudio software. Individual-based CNV calling was performed using the default parameters of the HMM model by integrating Log R Ratio, BAF, population allele frequency and the SNP distance.

To reduce the false discover rate in CNV calling, we used a calling criteria requiring that the standard deviation (SD) of LRR must be under or less 0.35, the CNV contained three or more consecutive SNPs and the length of CNV region must be more than 50 kb at the calling-level. We set the “-qcnumcnv 50” argument in the command line to treat any samples with > 50 CNV calls as low quality samples and eliminated them from analysis. GC model file was used to adjust the signal intensity values for CNV calling. For F_2_ individuals, the “-trio” argument was employed in CNV calling to make use of the family information. The CNVs whose 50.0% of sequence overlapped with the telomere region and those detected in only one individual and not overlapped with any other discovered CNVs were also removed from analysis. All putative CNVs identified in this study were pooled across breeds. We aggregated the overlapping CNVs identified across all samples to determine CNVRs following the previously published protocols [2].

### Quantitative real time PCR

Seven CNVRs were randomly selected for validation by quantitative real time PCR (qPCR). The 2^-△△Ct^ method was used to estimate relative quantification (RQ) of CNVRs [64]. This comparative method used a target assay for the CNV region and a reference assay of *β-ACTIN* as an internal control. The test and control primers were verified for their amplification efficiency. Six DNA samples were randomly selected including those with or without copy number variant for each CNVR. Primers and TaqMan probes labelled with FAM for each CNVR were designed with Allele 6.0 software (Applied Biosystems, Foster City, USA) and are listed in Additional file 2: Table S3. qPCR was carried out in a total volume of 20 μl mixture containing 1 × Premix Ex Taq™ (TaKaRa, Dalian, China), 0.2 μmol/L each primer, 1 × ROX Reference Dye II and 100 ng genomic DNA in an ABI 7500 FAST instrument (Applied Biosystems, Foster City, USA). The thermal cycle parameter was: 30 sec at 95°C, and 40 cycles of 3 sec at 95°C and 30 sec at 60°C. Each sample was analyzed in triplicate. Results were analyzed with the ABI7500 software v2.0.5 (Applied Biosystems, Foster City, USA).

### Gene identification and functional classification

Genes within porcine CNVRs were annotated by BioMart [32]. These genes were tested for enrichment of molecule function, cell component and biological process in gene ontology (GO) terms in DAVID Bioinformatics Resources 6.7 [65]. Considering the limited number of pig genes assigned to GO terms, the human annotated genes that were homologous to pig genes were used as the background. Multiple tests were corrected by FDR corrections and enrichment threshold was set as EASE score of adjusted FDR *P* ≤ 0.05.

### Statistical analysis

Association of the CNV268 with MCH and MCV was analyzed with a mixed linear model. Gender and batch were considered as fixed effects. The QTL for MCH and MCV was re-mapped with the CNV268 as the fixed effect. All the analyses were performed with R package.

## Abbreviations

LD: Logissimus muscle; SOX5: Sex determining region Y-box 5; STX17: Syntaxin 17; FUT1: Alpha-1-fucosyltransferase; APOE/C1/C4/C2: Apolipoprotein E/C1/C4/C2; KIT: V-kit Hardy-Zuckerman 4 feline sarcoma viral oncogene homolog; HADHA: Hydroxyacyl-CoA dehydrogenase, alpha subunit; HADHB: Hydroxyacyl-CoA dehydrogenase, belta subunit; KDR: Kinase insert domain protein receptor; ANP32B: Acidic (leucine-rich) nuclear phosphoprotein 32 family, member B; BSCL2: Berardinelli-Seip congenital lipodystrophy 2 (seipin); LTBP3: Latent transforming growth factor beta binding protein 3; GDF3: Growth differentiation factor 3; GYS1: Glycogen synthase 1; CAV1: Caveolin 1.

## Competing interests

The authors declare that they do not have any competing interests.

## Authors' contributions

LSH, conceived and designed the experiments, revised the manuscript; CYC, performed the experiments, analyzed the data, wrote and revised the manuscript; RMQ, detected CNVs by PennCNV, performed the q-PCR assays and wrote part of draft. RXW: analyzed the data. JR, provided comments and suggestions for the manuscript; JWM: genotyped the F_2_ individuals with 60K SNP chip. HAS: mapped the SNPs to reference genome; YMG: analyzed the data. All authors read and approved the final manuscript.

## Supplementary Material

Additional file 1 **Table S1.** CNVRs identified in this study and its state. Click here for file

Additional file 2 **Table S3.** Primers and probes used for qPCR assays. Click here for file

Additional file 3 **Figure S1.** The schematic diagrams depicting the validation of 7 CNVRs by quantitative real time PCR. Click here for file

Additional file 4 **Table S2.** The distribution of CNVs and CNVRs in each of 18 diverse populations. Click here for file

Additional file 5 **Table S4.** 1,055 well annotated genes located within the identified CNV regions. The 634 genes marked by * are also the copy number variable genes in human. Click here for file
