# Supplementary material for: A comprehensive survey of copy number variation in 18 diverse pig populations and identification of candidate copy number variable genes associated with complex traits
Source: BMC Genomics. 2012 Dec 27;13:733. doi: 10.1186/1471-2164-13-733 (PMC3543711; doi:10.1186/1471-2164-13-733)

**Figure legends**

**Figure S1. The schematic diagrams depicting the validation of 7 CNVRs by quantitative real time PCR**

The schematic diagrams ( A～G ) depict the validation results of 7 CNVRS by qPCR. The horizontal dashed line represents the relative quantification (RQ) value of the reference animal. Each bar represents the relative copy number in comparison to the reference individual. The X-axis indicates the sample ID, and the Y-axis shows RQ value obtained by qPCR. Vertical bars represent the standard error. The samples whose ID were only composed of Arabic number were from White Duroc × Erhualian F_2_ population. Individuals marked “S + Arabic number” were from Shaziling × Erhualian F_2_ population, T24 from Tongcheng × Erhualian F_2_ population, R44 and R72 from Rongchang × Erhualian F_2_ population, RC274 and RC313 from Rongchang breed, JH177 from Jinhua breed, NCYZ9 from wild boars.

1. CNVR329: Individual S139 was false positive. Individual S120 was reference sample. Individuals R44, S44, S45, S120 and S140 were confirmed.
2. CNVR361: Individuals 1912, 721 and 920 were false positive. Individual 920 was reference sample. Individuals 1790, 1792 and 683 were confirmed.
3. CNVR482: Individual 1440 was false positive. Individual 1031 was reference sample. Individuals 1133, 1790, 621, 920 and 1031 were confirmed.
4. CNVR531: Individuals 535 and 769 were false positive. Individual 621 was reference sample. Individuals 1031, 1440, 75 and 621 were confirmed.
5. CNVR419: Individuals 1790 and 1792 were false positive. Individual 1790 was reference sample. Individuals 1912, 1031, 621 and 721 were confirmed.
6. CNVR509: Not confirmed.
7. CNVR502: Individuals 134, 158, 625 and 82 were false positive. Individual 106 was reference sample. Individuals 106 and 1746 were confimed.


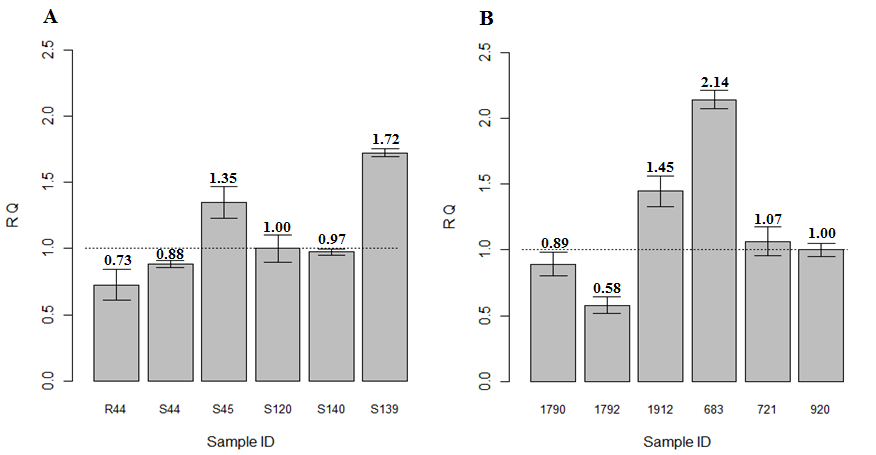


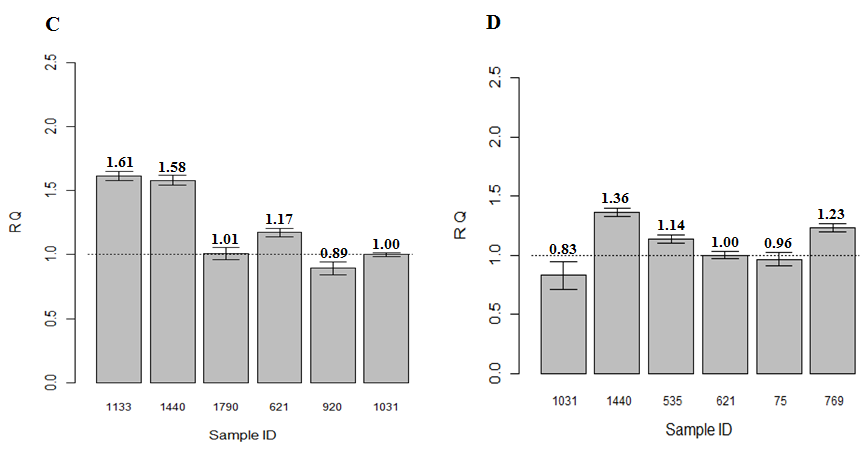


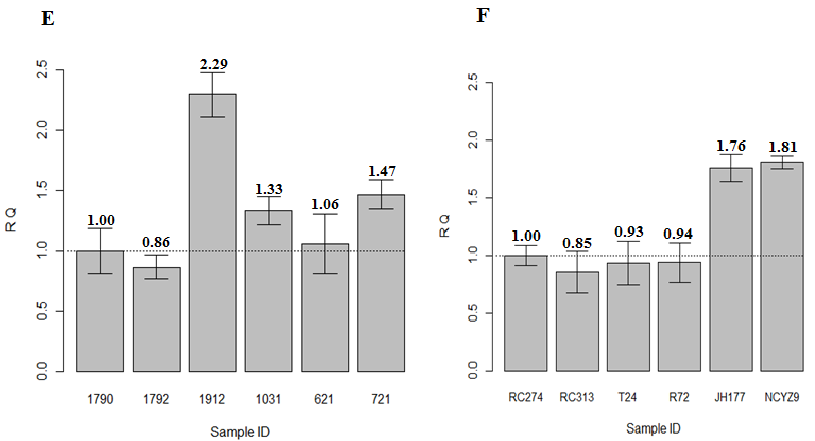


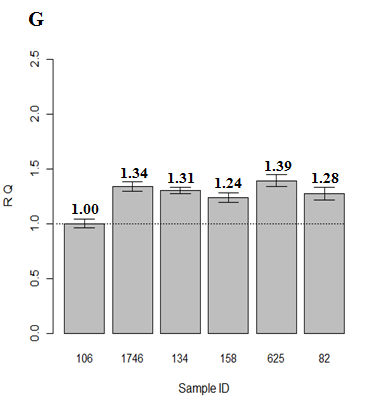

Supplement: Additional file 3 — Figure S1. The schematic diagrams depicting the validation of 7 CNVRs by quantitative real time PCR. [file 1471-2164-13-733-S3.doc]
